# Information Accrual From the Period Preceding Racket-Ball Contact for Tennis Ground Strokes: Inferences From Stochastic Masking

**DOI:** 10.3389/fpsyg.2019.01969

**Published:** 2019-08-27

**Authors:** Sepehr Jalali, Sian E. Martin, Tandra Ghose, Richard M. Buscombe, Joshua A. Solomon, Kielan Yarrow

**Affiliations:** ^1^Department of Psychology, City, University of London, London, United Kingdom; ^2^Department of Psychology, Technische Universität Kaiserslautern, Kaiserslautern, Germany; ^3^School of Health Sport and Bioscience, University of East London, London, United Kingdom; ^4^Centre for Applied Vision Science, City, University of London, London, United Kingdom

**Keywords:** sports, tennis, occlusion, reverse correlation, anticipatory ability

## Abstract

Previous research suggests the existence of an expert anticipatory advantage, whereby skilled sportspeople are able to predict an upcoming action by utilizing cues contained in their opponent’s body kinematics. This ability is often inferred from “occlusion” experiments: information is systematically removed from first-person videos of an opponent, for example, by stopping a tennis video at the point of racket-ball contact, yet performance, such as discrimination of shot direction, remains above chance. In this study, we assessed the expert anticipatory advantage for tennis ground strokes via a modified approach, known as “bubbles,” in which information is randomly removed from videos in each trial. The bubbles profile is then weighted by trial outcome (i.e., a correct vs. incorrect discrimination) and combined across trials into a classification array, revealing the potential cues informing the decision. In two experiments (both with *N* = 34 skilled tennis players) we utilized either temporal or spatial bubbles, applying them to videos running from 0.8 to 0 s before the point of racket-ball contact (cf. Jalali et al., 2018). Results from the spatial experiment were somewhat suggestive of accrual from the torso region of the body, but were not compelling. Results from the temporal experiment, on the other hand, were clear: information was accrued mainly during the period immediately prior to racket-ball contact. This result is broadly consistent with prior work using nonstochastic approaches to video manipulation, and cannot be an artifact of temporal smear from information accrued after racket-ball contact, because no such information was present.

Elite athletes demonstrate extraordinary ability in their sport of choice. While their sporting acumen may seem like a fundamentally physical attribute, it is in fact scaffolded by a range of cognitive skills that span the sensorimotor pipeline, from perception to action execution (Yarrow et al., 2009). One such skill that has received considerable attention from experimental psychologists is the expert anticipatory advantage.

The expert anticipatory advantage in sports describes a domain-specific benefit that sportspeople exhibit when predicting what is about to happen based on their opponent’s current bodily kinematics (as opposed to their opponent’s previous action history, which provides a separate cue for predicting current behavior; Mann et al., 2014). This advantage has been demonstrated in experiments simulating a variety of sports, most commonly via temporal and spatial occlusion methodologies (e.g., Jones and Miles, 1978; Abernethy, 1988). Hence, the advantage is widely exhibited, although the extent to which it benefits actual competitive performance remains uncertain (van Maarseveen et al., 2018).

A typical occlusion experiment runs as follows. A sporting scenario is selected, for example, a football (soccer) goalkeeper attempting to save penalties (e.g., Dicks et al., 2010; Smeeton and Williams, 2012). Videos are shot from the sportsperson’s (here, the goalkeeper’s) perspective, capturing various instances of two or more categories of outcome (for example, penalties struck to the left or right of the goalkeeper). In the actual experiment, participants, often varying in sports expertise (e.g., novice vs. expert goalkeepers) view these videos, attempting to discriminate which outcome will occur on each trial. Critically, the videos are manipulated to exclude some of their visual information. In temporal occlusion, the video is usually terminated early (for example, at or before ball contact), so that only particular sequences of body kinematics are available to guide the response. In spatial occlusion, particular features at constrained spatial locations (for example, the striker’s hips) are also removed from the video.

The logic of these experiments is that participants will only be able to perform at above-chance levels if there is information in the video to guide their decision, with performance declining toward chance as this information is systematically removed. Certain sports, such as cricket, have been long-running favorites in the occlusion literature (e.g., Abernethy and Russell, 1984; Müller and Abernethy, 2006; Müller et al., 2006), but occlusion approaches have been applied to sports as diverse as volleyball (e.g., Loffing et al., 2015) and karate (Mori et al., 2002).

Racket sports (e.g., badminton and squash; Abernethy and Russell, 1987; Abernethy, 1990) have been particularly well studied via occlusion techniques. The focus of the current study is the sport of tennis. This sport was among the first to provide evidence of an expert anticipatory advantage, with Jones and Miles (1978) showing that experts were above chance (and better than intermediate or novice players) at guessing the landing position of a serve when the video was stopped 0.042 s before ball contact. Subsequent work has found, for example, that experts extract information from the time when the ball’s toss is at its apex onward when predicting spin (Goulet et al., 1989). The temporal occlusion method has also been adjusted slightly to present one of several possible windows of visibility (0.3 s in duration) during service, with above-chance performance for experts when viewing the video for only the 0.3 s immediately before ball contact (Farrow et al., 2005). These temporal occlusion results are supplemented by spatial occlusion studies showing that, for example, experts can still discriminate the direction of tennis serves at above-chance levels following removal of body regions such as the entire lower body, but not when the ball’s toss was occluded (Jackson and Mogan, 2007). Experts were also impaired (but to a lesser extent) by removal of the arm and racket.

While the tennis serve is the most straightforward scenario to investigate, ground strokes have also been probed via occlusion methods. With temporal occlusion at ball contact, experts were above chance to discriminate between left/right lobs and passing shots when shutter goggles were used to block vision *in situ* on a tennis court (Shim et al., 2005). More traditional video-based studies have shown that unlike novices, experts could already predict shot direction above chance at −0.12 s relative to ball contact, with further improvements for occlusion occurring at −0.08 and −0.04 s (Rowe et al., 2009). Spatial occlusion work suggests that the arm/racket regions are critical when predicting ground-shot direction (Shim et al., 2006).

Video-based occlusion methods are not perfect, and our knowledge about the expert anticipatory advantage has been supplemented by a variety of techniques. Such techniques include eye tracking to provide information about where sportspeople attend, and animating/manipulating the opponent (e.g., Cañal-Bruland et al., 2011; Ida et al., 2013) including via virtual reality (Vignais et al., 2015). For example, Ida et al. (2013) manipulated the arm/racket angles of computer-generated opponents to successfully influence experts’ analogue estimates of the direction, speed, and spin of a tennis serve. In another study, swapping the arm/racket of stick-man representations of an opponent to that of a different shot confused experts trying to predict the direction of ground strokes (Cañal-Bruland et al., 2011). However, here we stay closer to the traditional occlusion approach, but attempt to remedy a possible weakness of the method: its dependency on experimenter decisions regarding exactly what to occlude.

To this end, we utilize a stochastic method of video occlusion borrowed from the psychophysical literature (Ahumada and Lovell, 1971), specifically a form of classification-image analysis (sometimes called reverse correlation) known as bubbles (Gosselin and Schyns, 2001). Bubbles are Gaussian-profiled windows of visibility that reveal the information from an otherwise masked (e.g., uniform gray) display. In the temporal domain, they are rather like the occlusion approach of Farrow et al. (2005), who displayed only a 0.3 s window of information from a video at a time. However, unlike in that study, which utilized a discrete set of nonoverlapping windows as separate conditions, in a bubbles experiment, several bubbles typically appear on each trial and the midpoint of each bubble is chosen at random. Furthermore, their Gaussian profiles remove transients and give the impression of the underlying display being smoothly revealed and subsequently re-masked (see Figure 1, for illustration). At the analysis stage, the random bubbles profiles from the different trials are binned by correctness of response and combined to produce a classification sequence. This classification can then be used to highlight the regions from which information must have been utilized to generate correct discriminations.

**Figure 1:**
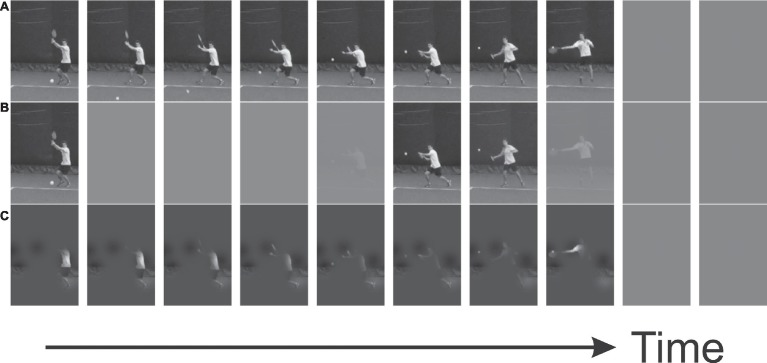
Example stimuli, shown as snapshots from video every 100 ms. **(A)**. Video occluded at point of racket-ball contact but with no bubbles manipulation (equivalent to pretest trials here). **(B)**. Temporal bubbles permit viewing of entire image, but only at certain times. **(C)**. Spatial bubbles permit viewing of only certain regions of the image, but across all (precontact) frames.

Although bubbles are typically applied to sparse, tightly controlled psychophysical stimuli, their applicability to a complex real-word scenario like tennis anticipation has been demonstrated recently (Jalali et al., 2018). In that study, we had both novice and competent tennis players view opponents in both service and forehand-groundstroke scenarios. We did not stop the video at racket-ball contact, but the structure of the experiment encouraged participants to respond as quickly as possible while maintaining an acceptable level of accuracy. The bubbles technique proved effective in both the temporal and spatial domains, but it suggested that our participants were primarily utilizing information from the beginning of the ball’s trajectory off the racket face rather than their opponent’s precontact kinematics. However, the temporal classification sequence did imply possible information accrual just prior to racket-ball contact as well, but this interpretation remained speculative. The reason is that the bubbles technique yields a classification sequence in which very discrete information sources can become smeared (i.e., exaggerated in extent), such that an information source at or just after racket-ball contact might spread back to appear significant in the immediately preceding frames.

Here, we again use bubbles to attempt to find evidence of an expert anticipatory advantage in tennis. Our aim is to quantify the extent of the temporal and spatial regions, prior to ball contact, from which skilled tennis players are able to extract useful information about shot direction, but using a stochastic masking technique (i.e., bubbles). The implementation of the bubbles method does not require any intuitions about information sources, which need to be designed as separate conditions, but rather allows any region of information to emerge in a bottom-up manner. As such, we believe it provides a useful form of methodological triangulation relative to traditional occlusion approaches. However, we made an important change relative to our previous study: We stopped the video at racket-ball contact, with bubbles appearing at random up to that point but no information ever provided afterwards. This change guarantees that any information sources we identify, even if near the point of racket-ball contact, are not the result of the aforementioned temporal smear arising at the analytic stage. We also focus on ground strokes only, without considering services. To presage our results, we find unequivocal evidence for the utilization of kinematic information by competent tennis players, but only for the period immediately prior to ball contact.

## Materials and Methods

### Participants

We utilized a smorgasbord^1^ sampling method, attempting to recruit participants with experience playing competitive tennis by various means. Where possible, we recorded their years of experience, current competitive tennis matches per year, and International Tennis Number (ITN), which is an index of their standard of play and ranges from ITN 1 (a player with extensive professional tournament experience and who currently holds or is capable of holding an ATP/WTA ranking) to ITN 10 (a player that is just starting to play competitively). Eleven participants (8 male, 3 female, mean age 30, mean years of tennis experience 13, mean matches per year 48, mean ITN 2.8) were recruited via adverts at London tennis clubs and by word of mouth, and traveled to City, University of London to participate. All completed both temporal and spatial bubbles sessions (see design, below)^2^. We also took the opportunistic step of developing a portable setup and taking it to the National UK University championships, where we recruited participants in their down time between matches (or after they had been eliminated). We tested 22 such participants in total, with 13 completing a spatial bubbles session (8 male, 5 female, mean age 22, mean years’ experience 11, mean matches per year 37, mean ITN 2.1) and 13 completing a temporal bubbles session (8 male, 5 female, mean age 22, mean years’ experience 10, mean matches per year 44, mean ITN 2.1)^3^. We subsequently took our portable setup to a second lab (at Technische Universität Kaiserslautern) in order to exploit its proximity to an elite school for sport (Heinrich Heine Gymnasium) attended by promising young tennis players and their coaches. We tested 10 such participants (8 male, 2 female, median age 16) who completed both spatial and temporal bubbles sessions^4^. For the German participants, we recorded their “Leistungsklassen” or performance class abbreviated as LK. According to the German Tennis Federation (DTB), the lowest class is LK23 and the highest LK1 consisting of top ranked players in Germany. The German pool had three LK1 players, one LK23 and average of LK 10 (std 8.5). They averaged 7.7 years of experience and 26 competitive matches per year. Finally, from the resulting complete samples of 34 (temporal bubbles)/34 (spatial bubbles) participants, we rejected participants, who were unable to perform the task significantly above chance during bubbles blocks (<55%, yielding binomial *p* > 0.05 that they were simply guessing), but only for our mean classification-array analysis (one of several analyses we ran; see below). We did this because an inability to perform the task makes it impossible for the bubbles technique to retrieve meaningful sources of information. This left final samples of 24 (spatial) and 27 (temporal) participants for mean classification-array analysis. Informed consent was obtained from all participants, who were paid £10 per h (London) and €10 per h (Germany) for their time. Ethical approval was granted by the relevant local Ethics Committees at City, University of London, and Technische Universität Kaiserslautern.

### Apparatus and Stimuli

We used the ground-stroke subset of video stimuli from those previously described by Jalali et al. (2018). They were recorded at a tennis club using a tripod-mounted camera (frame rate 120 Hz, frame size 1280 × 720 pixels). Four club coaches/hitters of a good but not elite standard acted as models and were instructed to “hit winners” without attempting explicit deception. They were situated near the baseline and recorded against a largely uniform blue backdrop. They were recorded playing forehand ground strokes (running rightward from a central position to return near the singles side line), directing their shots toward an imaginary receiver’s forehand or backhand. To increase image resolution, the camera was positioned at the net, on a line projecting from the filmed player to the imaginary receiver at the opposite baseline (height = 1.6 m, left of center line by 1.25 m).

Videos were first transformed to eight-bit grayscale. Two authors picked a subset of videos that were unambiguous (regarding the direction of the shot – line/cross), relatively homogeneous in terms of the position of the players at the time of ball contact, and lacking in artifactual cues that might allow the videos to be easily remembered for future classification (e.g., an unusual delivery trajectory). In each video, the frame corresponding to ball contact and the position at which the ball struck the racket head on this frame were manually identified for use in the subsequent presentation and analysis (see below).

The experiment was controlled by computers running scripts written in Matlab^®^ (The Mathworks, Natick, U.S.A.) using the Psychophysics Toolbox (Brainard, 1997; Pelli, 1997; Kleiner et al., 2007). Video stimuli were presented via either a cathode-ray tube monitor (for sessions at City, University of London), a short-throw gaming projector (Optoma^®^ GT760; for sessions at Kaiserslautern and temporal sessions at UK university championships), or a MacBook^®^ Pro (spatial sessions at UK university championships). The former two displays had a vertical refresh rate of 120 Hz, while the latter refreshed at 60 Hz, playing a down-sampled video. Only a central 600 × 400 pixel region of each video that excluded irrelevant peripheral information was presented. Displays were presented at around eye level and viewed at an appropriate distance in order to present the opposing tennis player with a height subtending ~4° visual angle (approximating their size as seen from the baseline during actual play). Participants responded by either stepping rightward or leftward, thus lifting the corresponding foot from one of two digital pedals, monitored at 100,000 Hz via a 16 bit A/D card (National Instruments X-series PCIe-6,323; for sessions at City) or by pressing an appropriate arrow key on a computer keyboard (all other sessions).

### Design and Procedure

There were two types of session incorporating either temporal or spatial bubbles blocks with participants completing one or both of these sessions, and in some cases up to two additional sessions not reported here (see footnotes 2–4). Each session took around an hour, and consisted of three blocks: One practice, one pretest, and one bubbles block (in that order). During practice, participants viewed small number of videos (between 10 and 24 depending on the experimental location; 50% to forehand, 50% to backhand) containing any of four players (8 possible videos per player) but with a preponderance of videos (70%) from one player and fewer videos (10% each) from the remaining three players, who were saved mainly for the experimental trials (see below). Videos were randomized with replacement.

Videos presentations began at −0.8 s relative to racket-ball contact. The practice block constituted a warm-up in which trials terminated at +0.2 s relative to racket-ball contact to provide clear information about the trajectory of the ball off the racket head. By contrast, in pretest and bubbles blocks, videos terminated at racket-ball contact (replaced with a uniform gray screen) or at the time of response if earlier than this.

For these pretest and bubbles blocks, 24 new videos (8 per player, 50% to forehand and 50% to backhand) were selected from the three players seen less often during practice. For the pretest, the videos were presented between one and four times each in a random order, yielding a block of either 24 trials (City and Kaiserslautern) or 96 trials (UK university championships). These differences reflected the fact that City and Kaiserslautern participants typically performed multiple sessions, hence, could have their pretest data combined across them. For the critical bubbles block, these videos were presented further 16 times each in a random order, yielding a block of 384 trials. Participants responded without any deadline. Trials with presentation glitches, that is, where one or more frames were dropped after the −0.2 s time point, were re-randomized and repeated at the end of the block. Feedback about correctness was provided after every trial.

Importantly, during bubbles trials only, the videos were subjected to random masking via the application of bubbles [see Figure 1; for videos showing examples of temporal and spatial bubbles, see videos 1 and 2, respectively from Jalali et al. (2018), available at https://www.frontiersin.org/articles/10.3389/fpsyg.2018.02229/full#supplementary-material]. Individual bubbles were combined to generate bubbles profiles in one (temporal) or two (spatial) dimensions. The number of bubbles presented began at 8 or 20 for temporal and spatial sessions, respectively. In principle, this (maximum) number could then be adjusted downward via a Quest staircase (Watson and Pelli, 1983), varying the number of bubbles in order to try and maintain participants’ performance at around 75% correct (i.e., lowering the number of bubbles if the task was too easy). However, as discussed further below, this was never required as the task was very hard even in the absence of any masking. The profile of each individual bubble was that of a 1 or 2-dimensional Gaussian density function, scaled to have unit height. In the temporal sessions, its width (*σ*) was 3 frames; in the spatial sessions, its width was 12 pixels (vertically and horizontally)^5^.

Bubble mean positions were selected at random within a domain extending throughout the relevant space of the video. Bubbles profiles were determined by combining the individual bubbles together. This was achieved by first reflecting bubble magnitudes around 0.5, then multiplying them together, and finally re-reflecting:

(1) $$\mathrm{Bubbles}=1-\overset{B}{\underset{b=1}{\prod }}\left(1-{\mathrm{bubble}}_{b}\right)$$

Pixel intensities were then calculated for display as the mean pixel intensity plus the difference between original and mean intensities multiplied by the Bubbles profile at each point. Expressed in terms of Weber contrasts, pixels were displayed at their original Weber contrasts multiplied by the Bubbles profile.

### Data Analysis

The saved Bubbles profiles from each trial formed the starting point in generating classification sequences (temporal conditions) or images (spatial conditions), which reveal the regions from which information supporting a correct response has been extracted. We calculated these classification arrays as per our previous report (Jalali et al., 2018). First, for the spatial condition only, Bubbles were re-centered so that the profile (saved in video coordinates) was translated to a new coordinate frame, centered on the ball at the time of racket-ball contact. Next, for each participant, a weighted sum of (re-centered) bubbles profiles yielded the raw classification array. The sum weights profiles from correct trials positively and profiles from incorrect trials negatively:

(2) $$\;\;\mathrm{RCA}=\overset{C}{\underset{c=1}{\sum }}{\mathrm{Bubbles}}_{c}-\overset{I}{\underset{i=1}{\sum }}{\mathrm{Bubbles}}_{i}$$

However, in order to provide more intuitive values for visualizing and combining data across participants, raw classification arrays were normalized to a z-like format. This was achieved via a permutation approach. For each of 2,000 iterations, correct/incorrect labels were randomly re-assigned (without replacement) to individual trials. The means and standard deviations at each point (i.e., each frame and/or pixel) calculated over these 2,000 permutations were used to z-score the classification array. This yielded an array varying around zero with positive values indicating regions of possible information accrual.

In order to draw statistical inferences across large arrays while controlling familywise type 1 error appropriately, data from all participants who were able to perform the task at significantly above-chance levels during bubbles blocks were combined and assessed via both cluster and *t*_max_ (also known as pixel or single-threshold) corrected permutation tests. These methods, derived from the neuroimaging literature (Blair and Karniski, 1993; Nichols and Holmes, 2002) are standard approaches for solving the multiple comparison problems with large sets of potentially correlated and non-normal data. Our particular implementation is more fully described in Jalali et al. (2018).

We also addressed a prediction particular to the data collected in these experiments, which, unlike typical bubbles experiments, were derived from participants, who rarely achieved 75% correct in a two-choice discrimination. We reasoned that the variability in performance across participants might be utilized in statistical inference. Bubbles are most efficient with 75% correct performance (Gosselin and Schyns, 2001) and would be expected to become less efficient, and thus produce classification arrays more dominated by random noise, with lower levels of discrimination performance. We would therefore expect that for an information-carrying region, there should be a positive correlation across participants between the magnitude of the classification array at that point and discrimination performance. We tested this prediction in a manner exactly analogous to the cluster/*t*_max_ approach, but using Pearson’s *r*-statistic in place of Student’s *t*-statistic in order to formulate cluster and *r*_max_ corrected permutation correlations. Where *t*-based tests reveal significant regions of information, *r*-based tests reveal regions more successfully exploited by better participants. All reported *p* are two-tailed, unless otherwise noted.

## Results

### Pretests

In pretest trials, participants saw the videos without degradation, but terminating at the point of racket-ball contact. Pretests were identical in spatial and temporal sessions, and our samples were not fully overlapping between these experiments, so data were collated across all 43 unique participants. Participants showed some ability to discriminate the direction of tennis ground strokes in the absence of information about the ball’s trajectory off the racket head (mean proportion correct = 0.632, SD = 0.093) and they did so on average at a level significantly above chance: Modeling these binomial data in the most appropriate way [i.e., with a general linear mixed model (GLMM) with logistic link function, incorporating a random term for the intercept] revealed a fixed intercept term of 0.55, which differed significantly from zero, that is, the null hypothesis of scoring 50% correct (*t*_[42]_ = 9.25, *p* < 10^−10^). For the subsets of U.K. participants reporting ITNs (*N* = 18), years of playing experience (*N* = 31), or matches per year (*N* = 27), these variables each were entered as lone predictors in separate GLMMs but failed to significantly correlate with discrimination performance (all *p* > 0.29). However, matches per year did become a significant positive predictor of performance (odds ratio = 1.011, 95% CI 1.004–1.18, *t*_[24]_ = 3.28, *p* = 0.003) when an outlying participant (claiming 150 competitive matches per year) was excluded.

### Temporal Bubbles

**Figure 2:**
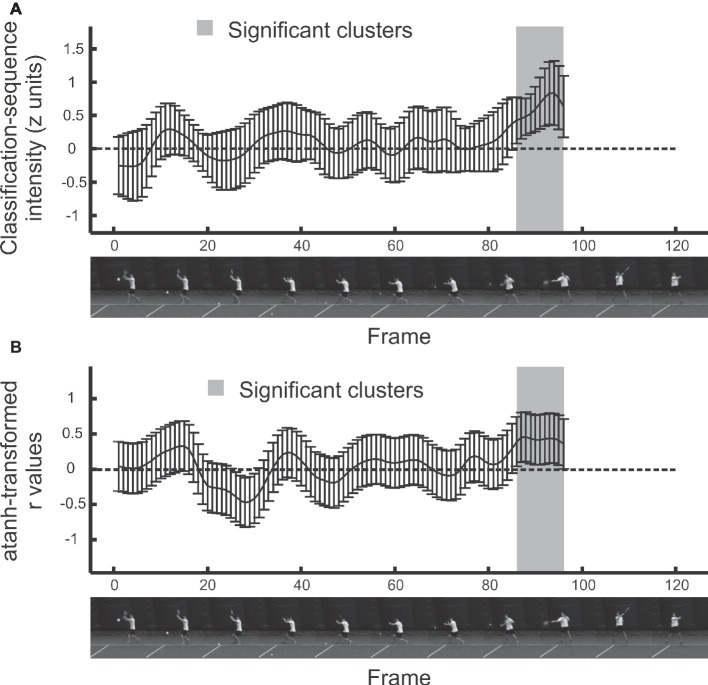
Results from temporal bubbles experiment. Error bars denote 95% confidence intervals. Shaded regions denote significant clusters. **(A)**. Mean *z*-scored classification sequence. **(B)**. Correlations between classification sequences and classification performance across participants.

In temporal bubbles trials, videos ran to the point of racket-ball contact, but only those periods revealed by randomly placed temporal bubbles were visible (Figure 1B). The Bubbles profiles from each trial were combined with accuracy data to create classification sequences for each participant. The mean *z*-scored classification sequence across participants is shown in Figure 2A, with positive values denoting regions from which information may have been extracted. No frames were significant after *t*_max_ correction, but a subset of frames (from 86 onwards, that is, from around 0.083 s before racket-ball contact) contribute to a significant cluster (*p* = 0.013). Cluster-based testing corrects for familywise error on the overall inference that the classification image differs reliably from zero, but does not imply that every point within the cluster is significant (Groppe et al., 2011), particularly in combination with the smoothing effects of bubbles (see Jalali et al., 2018, for further discussion). However, it is clear that some information was successfully extracted from the moment just before racket-ball contact.

Figure 2B shows additional results from a second statistical analysis. Here, instead of assessing the mean classification sequence for just those participants who were still able to perform above chance even during bubbles blocks, we assessed the correlation (for the entire sample of participants) between individual classification sequences and discrimination success. The raw values of *r* have been transformed to permit the creation of a constant confidence interval, which clarifies where possible clusters emerge. This happens wherever the confidence interval does not include zero, that is, for *r* values that are significant without any familywise correction. However, these transformed *r* values retain their basic meaning, in the sense that positive values represent frames where more successful participants (in terms of their ability to do the task) showed more positive classification sequence magnitudes. Our participants varied considerably in their ability to perform the task (between 50 and 75% correct). Because bubbles should be most effective (revealing pronounced peaks at points where useful information is extracted) for participants who approach 75% performance, and much less effective (reflecting mainly noise) for participants who are just guessing, these correlations are informative. Interestingly, the correlation analysis reveals a cluster with the exact same temporal extent as that found in the mean classification image (*p* = 0.029). Of course, these two analyses cannot be considered as independent tests. However, we believe they can sometimes be complementary to one another, as will become clearer in our spatial results.

### Spatial Bubbles

**Figure 3:**
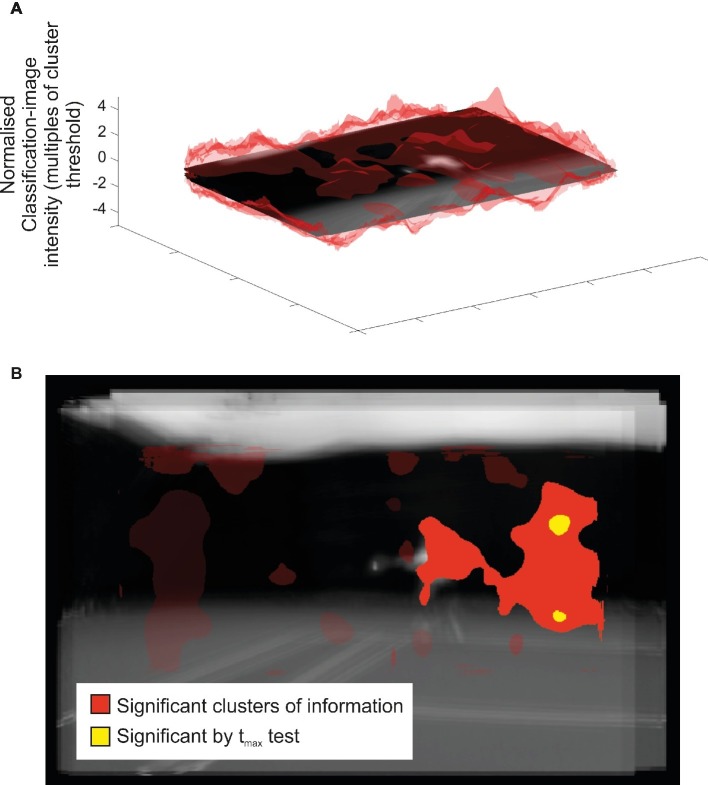
Classification image results from the spatial bubbles experiment. Results are overlaid on an image of the mean of all presented videos for the frames capturing racket-ball contact, centered on the point of racket-ball contact (hence constituent images do not perfectly align). However, the results of the spatial analysis are not specific to any one time point. **(A)**. Transparent red peaks denote mean classification-image intensity normalized to the cluster threshold value used in permutation testing (i.e., values more extreme than ±1 formed potential clusters). **(B)**. Solid colored regions were significant in cluster/*t*_max_ permutation testing, suggesting information might have been extracted from this part of the video. Transparent red regions denote nonsignificant clusters.

**Figure 4:**
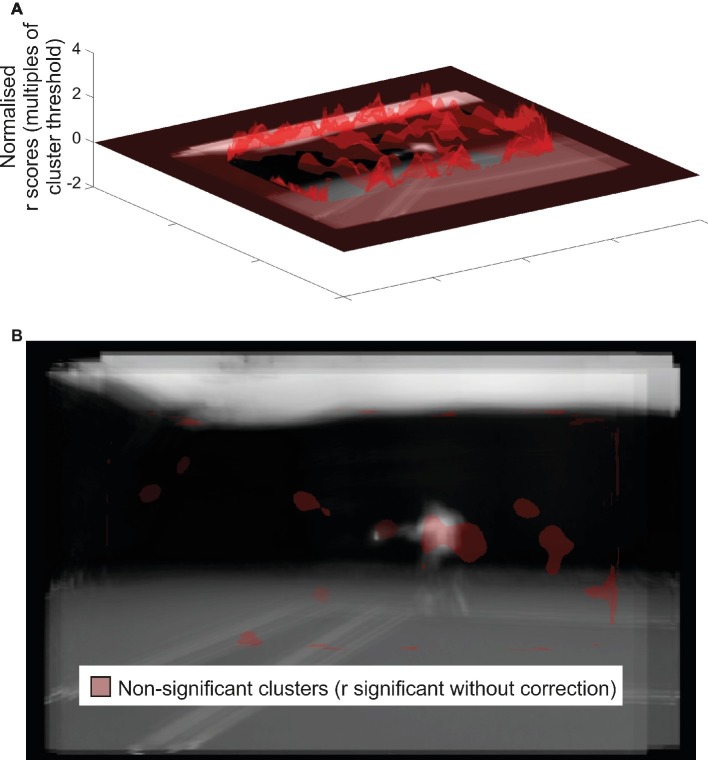
Correlation results from the spatial bubbles experiment. Results are overlaid on an image of the mean of all presented videos for the frames capturing racket-ball contact, centered on the point of racket-ball contact (hence constituent images do not perfectly align). However, the results of the spatial analysis are not specific to any one time point. **(A)**. Transparent red peaks denote correlations between classification-image intensities and discrimination performance, normalized to the cluster threshold value used in permutation testing (i.e., values more extreme than ±1 formed potential clusters). **(B)**. Transparent red regions denote points where the cluster threshold (representing a significant correlation in the absence of familywise correction) was exceeded, but resulted in only nonsignificant clusters.

In the spatial bubbles task, only particular areas of the video image were visible at random on each trial (Figure 1B). Data from our spatial bubbles experiment are shown in Figures 3, 4. Figure 3 shows the mean classification image, along with associated statistical inferences, for participants able to perform the bubbles task above chance. The top part of figure shows the classification image itself, while in the bottom part of the figure statistical thresholding has been applied to reveal a single large significant cluster (*p* = 0.0005). This cluster also incorporates two smaller regions that additionally survive *t*_max_ correction. This contrast *should* illustrate spatial areas from which visual information was accrued. However, the result is unconvincing. Although the cluster does include a region over the position of the opposing player’s body at the time of ball contact, this region only appears within the cluster by virtue of a slim connection to a larger and more pronounced region. The larger region might, at best, be considered to have overlaid parts of the opponent’s body at the beginning of the video, when they started their run to intercept the ball. However, this larger region would be inconsistent with the results of the temporal experiment, which suggested that useful information guiding the decision was not extracted until near the time of racket-ball contact.

Our complementary correlation-based analysis is shown in Figure 4, which in this case appears somewhat instructive. The format is the same as for the mean classification image shown in Figure 3 with the raw correlations shown at the top, and statistical thresholding applied at the bottom. However, in this case it is normalized correlation (*r*) values that are being illustrated and assessed for cluster or *r*_max_ based significance. No significant clusters were observed, but there is one nonsignificant cluster worthy of mention (one-tailed *p* = 0.096; all other clusters one-tailed *p* > 0.36) which sits over the position of the opponent’s body at the time of ball contact. This suggests a trend for those participants better able to discriminate shot duration during spatial bubbles sessions to have classification images that show stronger peaks in this region. In combination with the data from our analysis of the mean classification image (Figure 3), this result suggests that much (or all) of the cluster revealed there may represent a false positive, as it was no more likely to emerge in participants for whom bubbles had a good chance of actually working than it was for participants for whom bubbles could reveal only noise.

## Discussion

In our experiments, competent but nonelite tennis players first attempted to discriminate the direction of upcoming forehand ground strokes from videos of a tennis opponent, based only on information available prior to the point of racket-ball contact. On average, they were able to do so, in line with previous reports (Shim et al., 2006; Rowe et al., 2009). Unlike previous reports, we went on to remove additional information using a stochastic approach to video manipulation, by introducing bubbles rather than by applying systematic masking or image manipulation in a particular set of planned conditions. Our main finding was that participants used information from the period immediately before racket-ball contact, specifically within a window reaching back approximately 0.083 s, to perform the direction-discrimination task. Because this information source precedes racket-ball contact, it cannot include the trajectory of the ball off the racket head.

Our temporal results seem fairly consistent with previous reports. For example, Rowe et al. (2009) had tennis experts (broadly comparable to ours in competence, with ITNs of 2–4) judge forehand and backhand ground strokes (going to either the right or left) from videos which could be occluded at between −0.12 and +0.04 s relative to racket-ball contact. They found that experts could predict undisguised shot direction at approaching 75% correct when the video stopped at racket-ball contact, falling to around 60% when models were attempting disguise (c.f. 63% mean performance during pretest here; note that our models were instructed only to “hit winners,” but were presented to participants with smaller spatial extents than those of Rowe et al., to be more consistent with typical match viewing). Rowe et al. (2009) also found that experts could still discriminate the direction of ground strokes significantly above chance when the video stopped at either 0.12 or 0.08 s before racket-ball contact, but performed better with occlusion at 0 s. These results imply some accrual from roughly the temporal window we obtained here (in order to show improvement) but also some additional accrual from earlier frames (in order to still be performing above chance). Indeed, a similar study utilizing stick-man graphics in place of videos even found above chance performance with occlusion at −0.24 s, although performance actually then trended worse with occlusion at −0.16, −0.08, or 0 ms (Cañal-Bruland et al., 2011).

Our method was, in principal, well-suited to find the locus of any such early periods of information accrual, because bubbles could appear at any point back to 0.8 s before ball contact. Several possibilities should be considered regarding why we failed to find any such loci, reflecting the various limitations of our approach. The first relates to statistical power. Bubbles are a trial-hungry technique, with typical psychophysical applications using fairly simple stimuli and also very large numbers of trials (Gosselin and Schyns, 2001). This limitation is exacerbated when performance is only a little above chance even in the absence of any bubbles, as was the case here. Indeed, pretest performance suggests that our stimuli were very challenging to discriminate for most participants, so perhaps our stimuli simply did not contain usable information as early as the videos used in other studies, or perhaps it was sufficiently subtle that bubbles could not reveal it.

A second possibility is that information must be integrated over a protracted period, or combined from both of two temporally distinct epochs during early shot preparation, in order to be usable. Such temporally complex cues would still be present in standard temporal occlusion approaches where videos run continuously until a single occlusion point. However, while classification arrays can in principle reveal these kinds of features with enough trials, the bubbles approach is most efficient when the temporal extent of a cue is approximately matched to the temporal extent of an individual bubble (see for example, the simulations presented by Jalali et al., 2018). Note that various suggestions have been made within the bubbles literature to address this issue (Chauvin et al., 2005; Blais et al., 2012) and might be considered in future research on sports.

Regardless of whether there were any earlier information sources that went undetected in our experiment, we can at least assert with confidence that useful information was extracted from our videos immediately prior to racket-ball contact (although, as noted in the methods, we cannot assert that every individual frame highlighted by our cluster test was important). This ability may be learnt through regular match play, generalizing immediately to the particular opponents encountered here. It is also possible that the ability to anticipate was actually learnt entirely during the experiment, given that each stimulus was encountered multiple times. The correlation between pretest performance and matches per year suggests that more regular players are at least quicker to learn their new opponent’s kinematic “gives” (or perhaps they are quicker to learn other spurious cues in our videos, although we took steps to minimize these). However, this result must be considered tentative, as it was both exploratory, and relied on the exclusion of an outlying participant.

Our results from spatial bubbles sessions were not compelling and can at best be considered suggestive that our participants may have extracted some information from the torso region of their opponents. This would presumably be during the temporal window revealed by the temporal bubbles sessions, but the experiments are independent so this need not necessarily be the case. The need to apply statistical control across a much larger 2D space, relative to our temporal experiments, may have left our spatial experiment underpowered. We have previously shown that spatial bubbles can be effective with a setup and sample size similar to this one (Jalali et al., 2018), but in that case performance was nearer to 75% correct for all participants. Previous spatial occlusion work with video stimuli has been more conclusive. Shim et al. (2006) used a four-choice task (ground strokes or lobs to forehand or backhand), and found that removing the racket/arm impaired discrimination of videos when viewing was stopped at racket-ball contact. This suggests that these distal regions, which did not emerge in our analysis despite the fact that we centered our co-ordinate frame (and thus maximized power) at the racket head, are in fact important. However, they also observed performance which was still well above chance after these regions had been occluded. Therefore, participants must also have extracted information from other parts of the video, presumably proximal body segments, although the pattern of data was inconclusive in this regard. Indeed, some results from more recent studies using computer graphics in place of real videos suggest primacy for the proximal body: Fukuhara et al. (2017) found that an opponent rendered with a realistic body (but only point-light information for their arm and racket) was better predicted than one with a realistic arm and racket but only a point-light body.

In conclusion, we have replicated classic research showing that skilled tennis players can anticipate upcoming shots based on their opponent’s body kinematics. We also used a novel stochastic masking approach in order to highlight the role of the period immediately preceding racket-ball contact in supporting this ability. Although our bubbles approach could in principal have revealed a wider range of information sources relative to traditional occlusion studies (where a limited set of masking conditions must be selected in advance) in practice we have revealed, if anything, fewer such loci. The approach may still have merit, but primarily as a means of methodological triangulation, making an inference based on multiple complementary approaches, such as the temporal result observed here, more secure.

## Data Availability

The datasets generated for this study are available on request to the corresponding author.

## Ethics Statement

The studies involving human participants were reviewed and approved by Research Ethics Committee, City, University of London, and Research Ethics Committee, Technische Universität Kaiserslautern. Written informed consent to participate in this study was provided by the participants’ legal guardian/next of kin.

## Author Contributions

KY and JS conceived the experiments. SJ coded the experiments and analyses. SM, SJ, TG, and RB ran the experiments. KY drafted the manuscript. All authors contributed to the research design and critically revised the manuscript.

### Conflict of Interest Statement

The authors declare that the research was conducted in the absence of any commercial or financial relationships that could be construed as a potential conflict of interest.

## Footnotes

^1^This is our own dubious terminology. We originally intended to recruit several separate samples and address additional questions, but recruitment proved more challenging than expected, leading us to form a composite sample.

^2^Most of these participants also completed sessions in which they attempted to guess the direction of serves, but our service stimuli proved extremely difficult to discriminate, thus yielding no conclusive results, and are omitted from our report for concision.

^3^Nine from each group completed just a single block, and four competed both. Some participants failed to report some measures of experience, particularly ITN, so the means are based only on those who responded. Three participants from this group also completed a block using service stimuli, not reported here (see footnote 2).

^4^These participants completed two further blocks with a modified presentation sequence (a fixed rather than random ordering of opponents, to see if experiencing the same opponent repeatedly made them easier to predict) but this change did not generate any clear trend, and these blocks are not analyzed here.

^5^To speed calculations, each bubble was rounded to zero beyond 4 (temporal) or 3 (spatial) *σ* from its centre.
